# First-trimester Septic Abortion Due to *Salmonella enteritidis Oranienburg*

**DOI:** 10.1155/S106474499500010X

**Published:** 1995

**Authors:** LoriAnn Zettell, Russel D. Jelsema, Nelson B. Isada

**Affiliations:** 1 Division of Maternal-Fetal Medicine Department of Obstetrics and Gynecology Hutzel Hospital Wayne State University, 4707 St. Antoine Boulevard Detroit MI 48201 USA; 2 Division of Reproductive Genetics Department of Obstetrics and Gynecology Hutzel Hospital Wayne State University Detroit MI USA

## Abstract

*Background:* Septic abortion caused by transplacental salmonella infection is extremely rare; there are no reported cases of serotype *oranienburg* as an etiology.

*Case:* We describe a patient with non-typhoidal *Salmonella enteritidis* serotype *oranienburg* as a cause of first-trimester pregnancy loss. The rapid progression of this patient's septicemia and adverse outcome is described. The epidemiology and natural history of salmonella infections are also discussed.

*Conclusion:* Non-typhoidal salmonella is still a cause of morbidity in Western countries. This infection can result in rapid-onset fetal demise and septic abortion.


Infectious Diseases in Obstetrics and Gynecology 2:239–241 (1995)
© 1995 Wiley-Liss, Inc.

### First-Trimester Septic Abortion Due to *Salmonella enteritidis oranienburg*

**LoriAnn Zettell, Russel D. Jelsema, and Nelson B. Isada**

*Divisions of Maternal-Fetal Medicine (L.Z., R.D.J., N.B.I.) and Reproductive Genetics (N.B.I.), Department of Obstetrics and Gynecology, Hutzel Hospital, Wayne State University, Detroit, MI*

ABSTRACT

***Background:*** **Septic abortion caused by transplacental salmonella infection is extremely rare; there are no reported cases of serotype *oranienburg* as an etiology.**

***Case:*** **We describe a patient with non-typhoidal *Salmonella enteritidis* serotype *oranienburg* as a cause of first-trimester pregnancy loss. The rapid progression of this patient's septicemia and adverse outcome is described. The epidemiology and natural history of salmonella infections are also discussed.**

***Conclusion:*** **Non-typhoidal salmonella is still a cause of morbidity in Western countries. This infection can result in rapid-onset fetal demise and septic abortion.** © 1995 Wiley-Liss, Inc.

KEY WORDS

Salmonella, perinatal infection, pregnancy

A 22-year-old healthy black primigravida presented with a 24-h history of fever, chills, cramping lower abdominal pain, and lower back pain. She also described a cough beginning the previous week and diarrhea 1 day prior to admission. She had been seen earlier on the day of admission, and an ultrasound at that time confirmed fetal viability. Two hours later, she began having vaginal spotting and returned to the emergency department. She denied any attempts to terminate her pregnancy.

On admission, she was diaphoretic with a blood pressure (BP) of 102/62, pulse of 128, respiratory rate of 20, and temperature of 40.5°C. Her lungs were clear to auscultation. The abdomen was soft with suprapubic tenderness and absent rebound. A speculum examination revealed a closed os with a new yellow, purulent discharge, but no visible vaginal bleeding. A bimanual examination showed a 12-week-size uterus with marked cervical motion tenderness. The adnexa were unremarkable. No skin rash was present. A rectal examination was not performed.

An ultrasound revealed a single 12-week intrauterine pregnancy with no cardiac motion. The laboratory values showed a white blood cell (WBC) count of 22,900, hemoglobin of 9.4 gm/dl, and urinalysis of 20–50 red blood cells (RBC) with 20–50 WBC/high-powered field (hpf). Her coagulation values were normal.

A clinical diagnosis of septic abortion was made; ampicillin, gentamicin, and clindamycin were started. A dilatation and suction curettage (D&C) was performed 2 h after antibiotic administration. The uterine contents were not cultured. After the D&C, the patient's BP decreased to 70/30 and her aO2 saturation decreased to 80%. A chest roentgenogram revealed a left lower lobe infiltrate; erythromycin was added. She was admitted to the maternal special-care unit.

Her blood cultures were positive for a gram-negative rod subsequently identified as *Salmonella*

**Address correspondence/reprint requests to Dr. LoriAnn Zettell, Department of Obstetrics and Gynecology, Hutzel Hospital, 4707 St. Antoine Boulevard, Detroit, MI 48201.**

Received October 6, 1993
Accepted October 7, 1994

*enteritidis* serotype *oranienburg*. Chloramphenicol was started, and the other antibiotics stopped. The patient became afebrile after 3 days and her infiltrate resolved. The histology of the uterine contents revealed sclerosed and necrotic chorionic villi and fetal parts. The stool culture was positive for salmonella. Sputum and cervical cultures were negative. She was discharged home on hospital day 8 after remaining afebrile for 5 days.

Further history from the patient suggested a clean home environment with adequate plumbing. The patient ate scrambled eggs and worked as a lunch aide, but was not directly involved in handling food. No one else at home or work had been ill.

Her stool culture was positive 12 weeks after discharge but negative by 12 months. At the time of her most recent follow-up, she had a negative stool culture, was pregnant again, and went on to deliver a healthy term infant.

### DISCUSSION

### Epidemiology

In the vast majority of cases, human beings acquire salmonella from the ingestion of contaminated food or water. *Salmonella* has been isolated from almost all animal species including poultry, cows, pigs, and pets such as turtles, dogs, cats, and mice. Meat, especially beef and pork, is associated with 13% and dairy products with 4% of salmonella outbreaks. The majority of reported cases of salmonella infections in the United States are sporadic. Most cluster outbreaks are associated with poultry and poultry products, primarily eggs. An estimated 0.01% of all shell eggs contain *S. enteritidis*. Consequently, foods containing raw or undercooked eggs pose a risk for infection.[1]

Salmonella in the feces of infected hens may contaminate the surface of egg shells or reach the interior of the egg through hairline cracks. In hens with ovarian infection, the organisms may gain access to the yolk. The Eggs Product Inspection Act of 1970, which requires pasteurization of all bulk egg products and federally supervised inspection of egg shells for cracks, has been associated with a decline in egg-associated outbreaks, but salmonella persists as a source of infection. In 1985–89, of the 109 outbreaks of *S. enteritidis* in which a food vehicle was identified, 89 (82%) were associated with shell eggs.[2]

### Natural History

Enteric fever is a clinical syndrome classically produced by *S. typhi*. Enteric fever produced by *S. typhi* is typhoid fever, whereas that produced by other *Salmonella* serotypes is paratyphoid fever. Typhoid fever has decreased with the advent of good sanitation; however, morbidity arising from other serotypes has been increasing in recent years. In 1989, 8,549 *S. typhimurium* isolates were reported; historically, it has been the most frequently reported serotype, accounting for 21% of isolates. In the same year, 8,340 *S. enteritidis* cases represented 20% of all reported salmonella isolates.[2]

The incubation is usually 10–14 days (range 7–21 days) and is influenced by the number of organisms ingested. The onset is often insidious; the symptoms consist of fever, malaise, anorexia, headaches, and myalgias. Remittent fever is prominent. Either constipation or diarrhea may be present. Respiratory symptoms, including cough or sore throat, may be prominent. Rose spots are observed in up to 50% of patients. The abdomen is often tender and hepatosplenomegaly is noted in 25–50% of cases.

Therapy with chloramphenicol, ampicillin, or trimethoprim-sulfamethoxazole shortens the duration of illness from 3–4 weeks to 3–5 days. A definitive diagnosis is made on the basis of positive blood cultures, which are found in 8% of patients seen in the first week of illness and 20–30% of untreated patients as late as the third week of illness. Isolation of the organisms from the stool is strong presumptive evidence of infection.[3]

Any *Salmonella* serotype can produce a bacteremia characterized by a hectic febrile course lasting days to weeks. Localized suppurative infections in 10% of patients may persist as pneumonia, osteomyelitis, or meningitis. Salmonella infections pose special risks in pregnancy. In 1930, Villarama and Galang[4] reported 64 pregnant women with typhoid fever who were treated with supportive care only and noted fetal and maternal mortality rates of 60% and 26%, respectively. Twenty-nine percent of these fetuses would have been considered to be at a viable gestational age.

### CONCLUSIONS

The most likely source of infection in our patient is contaminated food, but any relation to her occupation remains unknown. It is striking to note that her fetus died within a 2-h period prior to admission. Symptoms of an ensuing bacteremia, positive stool cultures, and a purulent cervical discharge indicate seeding of the endometrium and transplacental passage of *Salmonella* resulting in the death of this fetus. The transplacental route for infection has been occasionally reported for other serotypes of *Salmonella.*[5–8] This patient also had a simultaneous pneumonia, most likely from the bacteremia.

Our patient was discharged home after 8 days of intravenous (IV) antibiotic therapy. She was afebrile during the last 5 days. Although the current recommendations are 14 days of antibiotics for *S. typhi,* we considered an 8-day therapy as adequate for this case of non-typhoidal infection. Close follow-up at 1 week showed a positive stool culture, so monthly complete blood counts (CBCs) and stool cultures were instituted, along with enteric precautions and follow-up with the health department. As mentioned previously, the patient cleared the infection spontaneously.

Salmonella as a cause of gynecologic morbidity has been reported sporadically. Salmonella as a cause of nongonococcal pelvic abscess formation was described by Saltzman et al.[9] in 1984. It has also been associated with second-trimester pregnancy losses following amniocentesis, as reported by Scialli and Rarick.[10]

This case further illustrates that *Salmonella* is still a cause of disease in industrialized nations and that it can result in rapid fetal demise and septic abortion. As suggested by both Ault et al.[8] and Scialli and Rarick,[10] salmonella should be included in the differential diagnosis of a patient presenting with high fever, respiratory and gastrointestinal symptoms, or abdominal pain.

### REFERENCES

1. Centers for Disease Control: Outbreaks of *Salmonella enteritidis* gastroenteritis—California, 1993. MMWR 42: 793–796, 1993.
2. Centers for Disease Control: Update: *Salmonella enteritidis* infections and shell eggs—U.S., 1990. MMWR 39: 909–912, 1990.
3. Riggall F, Salkind G, Spellacy W: Typhoid fever complicating pregnancy. Obstet Gynecol 44:117–121, 1974.
4. Villarama A, Galang JS: Typhoid fever in pregnancy. Phil Islands Med Assoc 10:311–315, 1930.
5. Amster R, Lessing JB, Jaffa AJ, Peyser MR: Typhoid fever complicating pregnancy. Acta Obstet Gynaecol Scand 64:685–686, 1985.
6. Dalaker K, Andersen B, Lovslett K: Septic abortion caused by *Salmonella enteritidis*. Acta Obstet Gynaecol Scand 67: 185–186, 1988.
7. Balek AP, Fothergill R: Septic abortion due to invasive *Salmonella agona*. Postgrad Med J 53:155–156, 1977.
8. Ault KA, Kennedy M, Muhieddine A, Seoud F, Reiss R: Maternal and neonatal infection with *Salmonella heidelberg:* A case report. Infect Dis Obstet Gynecol 1:46–48, 1993.
9. Saltzman DH, Evans MI, Robichaux AG, Grossman JH, Friedman AJ: Nongonococcal pelvic abscess caused by *Salmonella enteritidis*. Obstet Gynecol 64:585–586, 1984.
10. Scialli AR, Rarick TL: Salmonella sepsis and second trimester pregnancy loss. Obstet Gynecol 79:820–821, 1992.
